# *Wolbachia*-Conferred Antiviral Protection Is Determined by Developmental Temperature

**DOI:** 10.1128/mBio.02923-20

**Published:** 2021-09-07

**Authors:** Ewa Chrostek, Nelson Martins, Marta S. Marialva, Luís Teixeira

**Affiliations:** a Instituto Gulbenkian de Ciência, Oeiras, Portugal; b Department of Evolution, Ecology and Behaviour, University of Liverpool, United Kingdom; c Institut de Biologie Moléculaire et Cellulaire, Université de Strasbourg, Strasbourg, France; d Department for Biomedical Research, University of Bern, Switzerland; e Faculdade de Medicina da Universidade de Lisboa, Lisbon, Portugal; The University of Texas at Austin; Max Planck Institute for Marine Microbiology

**Keywords:** *Wolbachia*, *Drosophila*, virus, temperature, symbiosis, development

## Abstract

Wolbachia is a maternally transmitted bacterium that is widespread in arthropods and filarial nematodes and confers strong antiviral protection in Drosophila melanogaster and other arthropods. *Wolbachia*-transinfected Aedes aegypti mosquitoes are currently being deployed to fight transmission of dengue and Zika viruses. However, the mechanism of antiviral protection and the factors influencing are still not fully understood. Here, we show that temperature modulates *Wolbachia*-conferred protection in Drosophila melanogaster. Temperature after infection directly impacts *Drosophila* C virus (DCV) replication and modulates *Wolbachia* protection. At higher temperatures, viruses proliferate more and are more lethal, while *Wolbachia* confers lower protection. Strikingly, host developmental temperature is a determinant of *Wolbachia*-conferred antiviral protection. While there is strong protection when flies develop from egg to adult at 25°C, the protection is highly reduced or abolished when flies develop at 18°C. However, *Wolbachia*-induced changes during development are not sufficient to limit virus-induced mortality, as *Wolbachia* is still required to be present in adults at the time of infection. This developmental effect is general, since it was present in different host genotypes, *Wolbachia* variants, and upon infection with different viruses. Overall, we show that *Wolbachia*-conferred antiviral protection is temperature dependent, being present or absent depending on the environmental conditions. This interaction likely impacts *Wolbachia*-host interactions in nature and, as a result, frequencies of host and symbionts in different climates. Dependence of *Wolbachia*-mediated pathogen blocking on developmental temperature could be used to dissect the mechanistic bases of protection and influence the deployment of *Wolbachia* to prevent transmission of arboviruses.

## INTRODUCTION

The environment affects not only individual organisms but also the relationships between them (1). Numerous physical and biological factors have been described as important for insect-microbe symbioses. These include insect population density (2–4), nutrient availability (5, 6), and interactions between factors with either synergistic or antagonistic effects (7). Temperature is a major factor that affects several different host-microbe symbioses and is relevant to understand host and symbiont distributions and to predict future outcomes of interactions in a scenario of global warming (1, 8–18).

*Wolbachia* is a maternally inherited intracellular bacterium that infects a wide range of arthropods and some nematodes. This endosymbiont induces strong phenotypes in many of its hosts, which may contribute to its success in invading and being maintained in host populations. *w*Mel, the *Wolbachia* strain present in Drosophila melanogaster, confers strong protection against a wide range of RNA viruses, which can be a fitness benefit in nature (19, 20). This protection extends to nonnative Wolbachia-host associations, including in mosquito vectors of human disease (21). Recently, *Wolbachia* has become one of the most promising approaches to control dengue and Zika viruses in the wild. It has been shown that the release of *Wolbachia*-infected Aedes aegypti mosquitoes can reduce the number of dengue cases in both areas where dengue is endemic or nonendemic (22–26). Although the molecular mechanisms of *Wolbachia*-conferred antiviral protection are not yet known, identification of factors influencing protection can contribute to understanding the association of *Wolbachia* and hosts in natural populations, as well as inform *Wolbachia*-based field interventions.

*Wolbachia-*conferred antiviral protection is influenced by host and bacterial genetics. Different *Wolbachia* strains in the same host genetic background differ in protection (27–31). In general, differences in *Wolbachia* titers are correlated with differences in protection, with higher titers conferring higher antiviral protection (27–31). However, some *Wolbachia* strains do not provide protection despite high titers (32). Host genetic variation can also contribute to the strength of *Wolbachia*-conferred protection, as seen in Aedes aegypti (33).

Environmental factors also affect this symbiont-mediated protection, either through modulation of *Wolbachia* titer or by titer-independent mechanisms. A host diet rich in cholesterol reduces antiviral protection in Drosophila melanogaster (6). Similarly, antibiotic treatment reducing *Wolbachia* titer reduces the antiviral protection (34). Finally, temperature has been shown to modulate *Wolbachia-*conferred protection to parasites in an engineered *Wolbachia*-host association; in Anopheles stephensi, temperature and somatic *Wolbachia* infection determined Plasmodium titer in mosquitoes (14). In Aedes aegypti mosquitoes stably transinfected with *w*Mel, the comparison of one constant and two fluctuating temperature regimes showed that the antiviral protection is robust across conditions and is unlikely to be compromised in antiviral field trials (35). However, a more recent study indicates that at a high temperature, *w*Mel is less protective (36). This is associated with lower titers of *w*Mel in A. aegypti mosquitoes reared at high temperatures, which may also affect the strength of cytoplasmic incompatibility and the vertical transmission of *Wolbachia* (36–39). On the other hand, different temperature regimes also affect dengue transmission independently of *Wolbachia* (40).

Despite two studies tackling the effect of temperature on *Wolbachia*-conferred protection in artificial associations, the impact of temperature on antiviral protection in natural *Wolbachia-*host symbiosis remains unknown. However, it is known that temperature can affect the interaction between *Wolbachia* and Drosophila. For instance, higher temperatures lead to greater proliferation, and, in the case of the pathogenic variants *w*MelPop and *w*MelOctoless, higher cost, in D. melanogaster (8, 41–43). Similarly, lower developmental temperatures lead to lower titers of *w*Yak in Drosophila yakuba, which in turn increases imperfect vertical transmission of this *Wolbachia* strain (44). Lower temperatures also affect the fitness of flies with *Wolbachia* after reproductive dormancy (45). The effect of temperature on *Wolbachia*-related phenotypes may explain why different *Drosophila* species with different *Wolbachia* strains or variants prefer different temperatures (46–48). The geographic distribution of *Wolbachia* in D. melanogaster populations could be explained by its relative fitness effects under varying thermal conditions (49, 50). Antiviral protection could be a temperature-dependent fitness benefit, which would be balanced against the potential cost of highly protective high-titer symbionts.

Here, we tested how temperature affects *Wolbachia* protection against viruses in natural *Wolbachia-*Drosophila associations. First, we asked how different virus doses and postinfection temperatures affect *Wolbachia* densities and antiviral protection. Then, we dissected the effect of developmental temperature on *Wolbachia*-carrying Drosophila melanogaster. We show strong interaction between this environmental variable and *Wolbachia*-conferred protection against viruses.

## RESULTS

**FIG 1:**
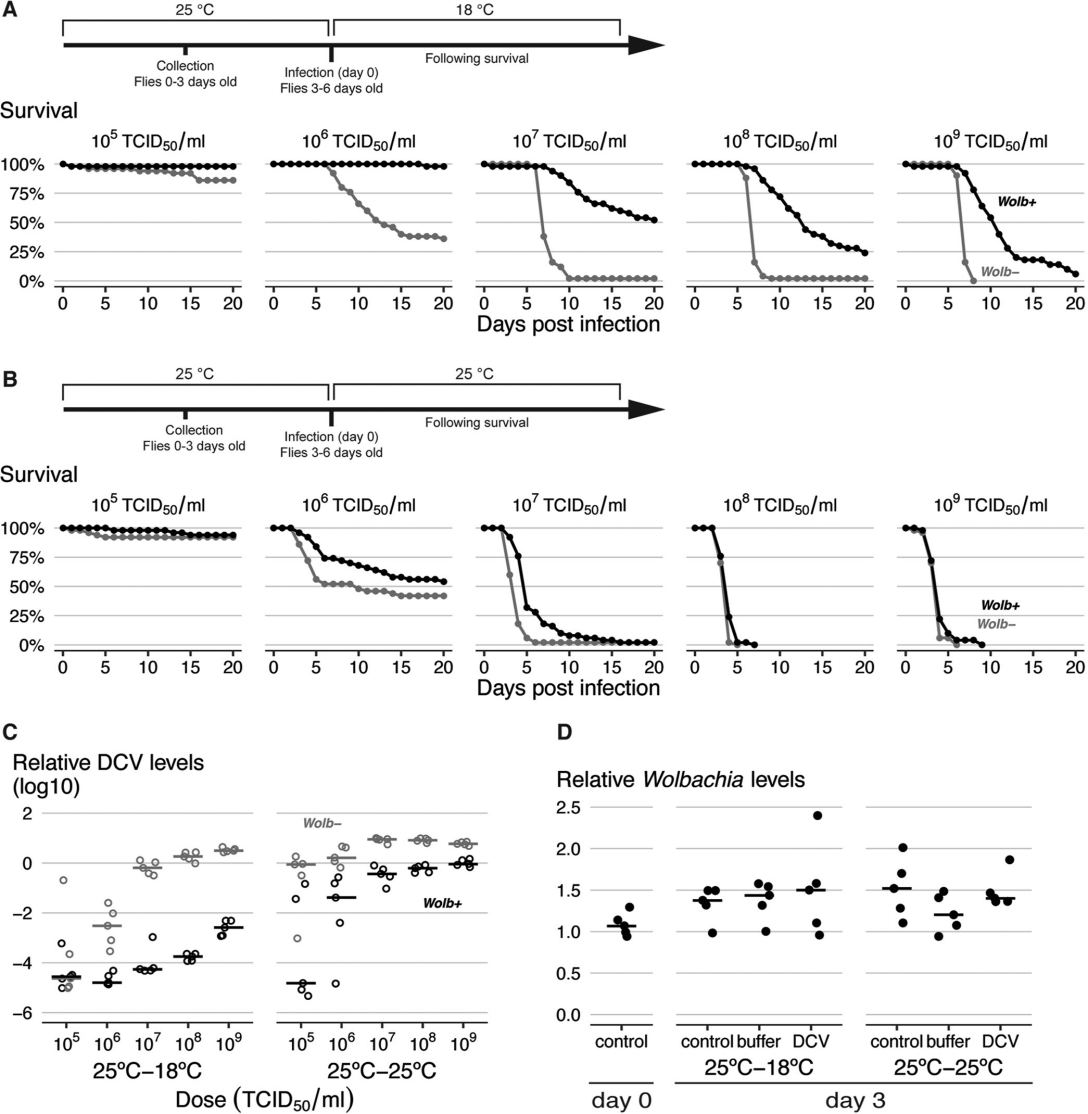
Postinfection temperature modulates the strength of *Wolbachia*-conferred antiviral protection. (A, B) Survival curves of flies infected at different temperatures, with the schemes of the experimental designs shown above. *Wolb^+^* and *Wolb^−^* flies, 50 per *Wolbachia* status and condition, were raised at 25°C, infected with different doses of DCV, and placed at either 18°C (A) or 25°C (B) after DCV infection. Mortality was recorded daily. (C) DCV titers in DCV-infected flies at 3 days postinfection (dpi) measured by quantitative reverse transcription-PCR (RT-qPCR). Flies were kept at 25°C before infection and at either 18°C or 25°C after infection. (D) *Wolbachia* levels measured by qPCR at the day of infection (3 to 6 days old), or at 3 days after in unchallenged (control), buffer-, or DCV-challenged (10^7^ TCID_50_/ml) flies. Flies were kept at 25°C prior to DCV infection and placed at either 18°C or 25°C after DCV infection. (C, D) Each dot represents a sample of 10 flies, and horizontal lines show medians.

To test how different infection temperatures affect *Wolbachia*-conferred protection to *Drosophila* C virus (DCV), we used the Drosophila melanogaster Drosdel *w^1118^* isogenic line (*iso*) carrying a natural *Wolbachia* variant, *w*MelCS_b (*Wolb*^+^), and a matching *Wolbachia-*free control (*Wolb*^−^) (19, 27). Flies were raised at 25°C, and 3- to 6-day-old flies were challenged with serial dilutions of DCV and subsequently placed at either 18°C or at 25°C (Fig. 1A and B). Virus-induced mortality was higher at 25°C than at 18°C at all doses except the lowest one, where there is almost no mortality associated with the infection (Cox hazard ratio [CHR], temperature effect, *P ≤ *0.001 for all comparisons, except with an infection dose of 10^5^ 50% tissue culture infective dose [TCID_50_]/ml). Importantly, *Wolbachia* protection against DCV varies with temperature (Wolbachia*temperature interaction effect, *P < *0.001), and it is stronger when the temperature at infection is 18°C (CHR = −1.84; *P < *0.001) rather than 25°C (CHR = −0.75; *P < *0.001). Overall, the temperature of infection affects the survival of the flies, and *Wolbachia* is more protective at a lower infection temperature (Fig. 1A and B).

To test if the interaction of *Wolbachia* protection with temperature is also reflected in viral loads, we measured viral titers in flies at 3 days postinfection (dpi) by quantitative reverse transcription-PCR (RT-qPCR) (Fig. 1C). We detected a strong interaction between *Wolbachia* presence, temperature of infection, and dose of the virus (linear model [LM], *P < *0.001 for the interaction). At the lower temperature, *Wolbachia* conferred more resistance at higher viral doses, as viral titers stayed very low at lower doses in both *Wolb^+^* and *Wolb^−^* flies. At the higher temperature, it conferred more resistance at the lower doses, as virus titers were very high in *Wolb^+^* and *Wolb^−^* flies exposed to high virus doses. The mean viral load was higher at 25°C than at 18°C (LM, *P < *0.001) and was lower in the presence of *Wolbachia* (LM, *P < *0.001). On average, *Wolbachia* induced higher resistance at 18°C than at 25°C. There was a 550-fold reduction in viral load at 18°C (LM, *P < *0.001) and a 50-fold reduction at 25°C (LM, *P < *0.001), producing an approximately 11-fold difference between these two conditions (LM, *P = *0.003). These results show that the strength of *Wolbachia*-conferred protection against DCV, in terms of both survival and viral loads, depends on the postinfection temperature. Protection is higher at a lower temperature. Since postinfection temperature affects viral infection independently of *Wolbachia*, the lesser capacity of *Wolbachia* to protect at higher postinfection temperatures may be related to higher replication rates of the virus.

However, antiviral protection is also usually positively correlated with *Wolbachia* levels (27, 28, 30, 34, 51, 52), so we tested *Wolbachia* levels at the day of infection and after 3 days in DCV-infected, buffer-pricked (control), or unmanipulated flies, at both temperatures (Fig. 1D). *Wolbachia* levels were not affected by virus, buffer, or temperature of infection (LM, *P > *0.425 for effect of temperature or treatment). Thus, the difference in protection is likely independent of *Wolbachia* levels, as these are not significantly affected within 3 days of the viral challenge.

**FIG 2:**
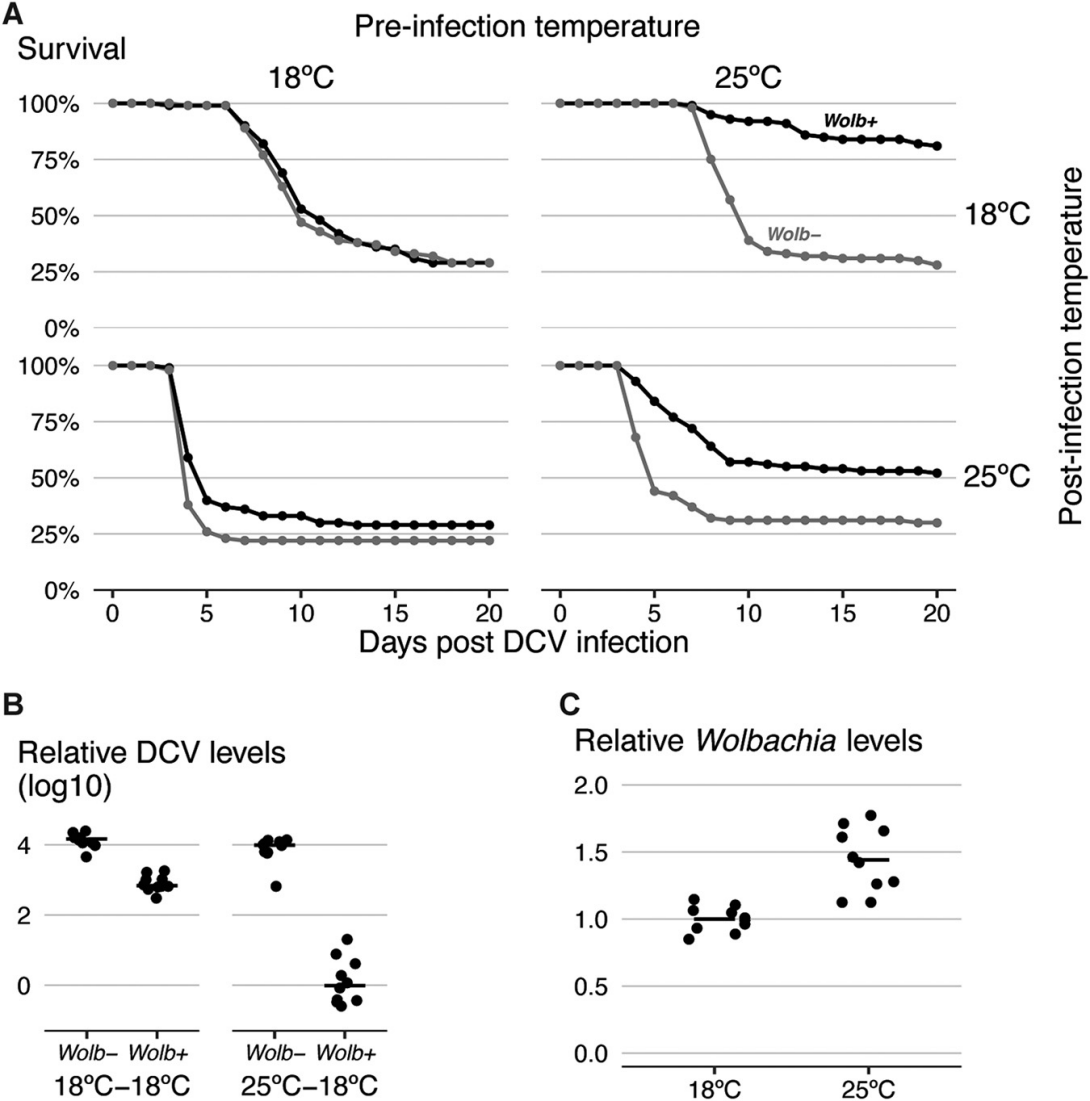
Preinfection temperature determines *Wolbachia*-conferred antiviral protection. (A) *Wolb^+^* and *Wolb^−^* flies, 50 per *Wolbachia* status per condition, were infected with DCV (10^8^ TCID_50_/ml) and checked for survival every day. Flies were raised and kept at 18°C or 25°C before DCV infection (preinfection temperature) and placed at either 18°C or 25°C after infection (postinfection temperature). (B) DCV titers in flies at 3 dpi measured by RT-qPCR. Flies were raised and kept at either 18°C or 25°C before infection and at 18°C after infection. A replicate of this experiment is shown in Fig. S1 in the supplemental material. (C) *Wolbachia* levels measured by qPCR in 3- to 6-day-old flies raised at either 18°C or 25°C. (B, C) Each dot represents a sample of 10 flies. Horizontal lines show medians of the samples.

We extended our analysis by testing how preinfection temperature, which includes that during egg laying, larval and pupal development, and the first days of adulthood, affects *Wolbachia* protection (Fig. 2). We followed the survival of infected flies under all four possible combinations of preinfection and postinfection temperatures of 18°C and 25°C (Fig. 2A). There is a strong interaction between preinfection temperature and *Wolbachia* (CHR, *P = *0.009). Remarkably, in flies raised at 18°C, *Wolbachia* does not protect against DCV infection (CHR, *P = *0.207), while in flies raised at 25°C it does (CHR = −1.38, *P < *0.001), irrespective of the postinfection temperature. Also, in the absence of *Wolbachia*, the preinfection temperature has no effect on the survival after DCV infection (CHR, *P = *0.276). This strong interaction between *Wolbachia*-conferred protection against DCV and preinfection temperature is also reflected in DCV loads (see Fig. 2B and Fig. S1 in the supplemental material; linear mixed-effects model [LMM], *P < *0.001). On average, *Wolbachia* reduced viral titers 14-fold at a preinfection temperature of 18°C and 1,900-fold at 25°C (LMM, difference between reductions, *P < *0.001). In these assays, preinfection temperature also did not affect viral loads in the absence of *Wolbachia* (LMM, *P = *0.534). Therefore, in contrast to postinfection temperature, preinfection temperature does not directly impact virus performance and modulates only *Wolbachia* protection. In summary, in flies raised at a lower temperature, *Wolbachia* confers reduced resistance to DCV in terms of viral titers and no protection in terms of survival.

10.1128/mBio.02923-20.1 FIG S1 Preinfection temperature determines viral titers. *Drosophila* C virus (DCV) titers measured by RT-qPCR at 3 days postinfection (dpi) in *Wolb^+^* and *Wolb^−^* flies infected with 10^8^ TCID_50_/ml DCV. Flies were kept at 18°C or 25°C before the infection and at 18°C after the infection. Each dot represents a sample, and each sample consists of 10 flies. Horizontal lines indicate medians of the replicates. This is a replicate of the experiment shown in Fig. 2B. Download FIG S1, TIF file, 1.4 MB. Copyright © 2021 Chrostek et al. 2021 Chrostek et al. https://creativecommons.org/licenses/by/4.0/ This content is distributed under the terms of the Creative Commons Attribution 4.0 International license.

Next, we measured *Wolbachia* levels in flies raised at 18°C and 25°C (Fig. 2C). Flies raised at 18°C had approximately 33% less *Wolbachia* than the flies raised at 25°C (LM, *P < *0.001). Although this difference may contribute to the difference in protection, a larger, 50% difference in *Wolbachia* titers between *w*Mel- and *w*MelCS-harboring flies did not abolish the protection (27). Thus, differences in *Wolbachia* titers do not seem to fully explain the difference in protection between flies raised at different temperatures.

**FIG 3:**
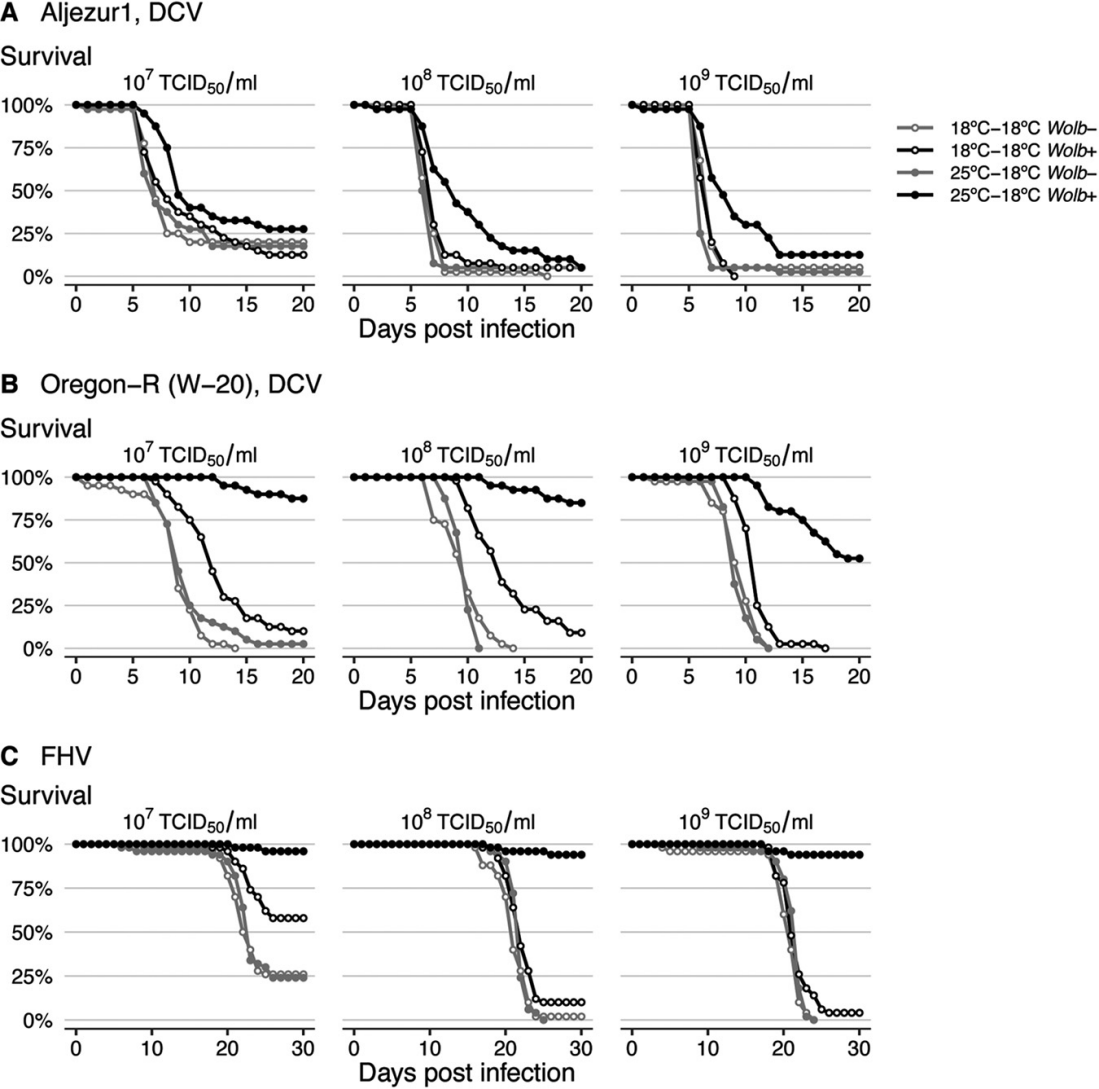
Preinfection temperature determines *Wolbachia*-conferred antiviral protection in different *Wolbachia* and *Drosophila* genotypes and against different viruses. (A) Aljezur 1 and (B) Oregon-R (W-20) *Wolb^+^* and *Wolb^−^* flies, 50 per *Wolbachia* status per condition, were pricked with three dilutions of DCV. (C) *w*^1118^ *iso* flies, 50 per *Wolbachia* status per condition, were infected with three doses of Flock House virus (FHV). Flies were kept at 25°C or 18°C before infection and at 18°C after infection. Mortality was recorded daily. A replicate of this experiment is shown in Fig. S2.

To test the robustness of preinfection temperature effect on *Wolbachia-*conferred protection, we tested flies with distinct host genetic backgrounds, Aljezur1 and Oregon-R W-20 (19), each harboring its original *w*Mel-like *Wolbachia* strain (Fig. 3A and B and Fig. S2A and B). In agreement with the previous results, there was a significant effect of preinfection temperature on *Wolbachia*-conferred antiviral protection against DCV in both lines (CHR, *P < *0.001 for both). *Wolbachia* in Aljezur1 line conferred protection only when the preinfection temperature was 25°C (*P = *0.763 for 18°C; CHR = −1.19, *P < *0.001 for 25°C). In the Oregon-R W-20 line, *Wolbachia* still conferred some protection when the preinfection temperature was 18°C, although it was significantly less than at 25°C (CHR = −1.21, *P < *0.001 for 18°C; CHR = −3.02, *P < *0.001 for 25°C; CHR difference = 1.8, *P < *0.001). Next, we investigated if the preinfection temperature effect was specific to DCV or if it was also present upon challenge with another RNA virus, Flock House virus (FHV) (Fig. 3C and Fig. S2C). There was an interaction between *Wolbachia*, preinfection temperature, and dose in this assay (CHR, *P = *0.013). *Wolbachia* protection was significantly higher in flies raised at 25°C for all doses of virus except for the lowest one (contrast of CHR < −2.84, *P < *0.001, for 10^7^ to 10^9^ TCID_50_/ml). At the lowest dose, 10^6^ TCID_50_/ml, there was an overall low mortality of flies, and the difference in survival was not statistically significant (contrast of CHR = −1.00, but *P = *0.421). The preinfection temperature had no effect on survival against FHV in the absence of *Wolbachia* (CHR, *P = *1, in all comparisons), as observed for DCV. In conclusion, the strong influence of preinfection temperature on *Wolbachia*-conferred protection is a general effect observed in different host genetic backgrounds, *Wolbachia* variants, and with the different viruses used.

10.1128/mBio.02923-20.2 FIG S2 Preinfection temperature determines *Wolbachia*-conferred antiviral protection in different *Wolbachia* and *Drosophila* genotypes and against different viruses. (A) Aljezur 1 and (B) Oregon-R (W-20) *Wolb^+^* and *Wolb^−^* flies, 50 per *Wolbachia* status per condition, were pricked with three dilutions of DCV. (C) *w*^1118^ *iso* flies, 50 per *Wolbachia* status per condition, were infected with three doses of Flock House virus (FHV). Flies were kept at 25°C or 18°C before the infection and at 18°C after the infection. Mortality was recorded daily. This is a replicate of the experiment shown in Fig. 3. Download FIG S2, TIF file, 1.6 MB. Copyright © 2021 Chrostek et al. 2021 Chrostek et al. https://creativecommons.org/licenses/by/4.0/ This content is distributed under the terms of the Creative Commons Attribution 4.0 International license.

Since there is a strong interaction between protection and constant preinfection temperature, we asked if protection exists when temperature is cycling daily between 18°C and 25°C (Fig. S3). *Wolbachia* protects against DCV infection at the cycling temperature (CHR = −0.67, *P = *0.014). This protection seems to be intermediate between the protection seen in flies developed at 18°C or 25°C. This result shows that under conditions of daily cycling temperatures, *Wolbachia* can protect against viruses.

10.1128/mBio.02923-20.3 FIG S3 *Wolbachia* protects flies kept at a temperature cycling daily between 18 and 25°C. Fifty *Wolb^+^* and *Wolb^−^* flies were raised with the temperature cycling daily between 25°C (midday) and 18°C (midnight), infected with DCV (10^8^ TCID_50_/ml), and placed at the same cycling temperature after infection. Mortality was recorded daily. Download FIG S3, TIF file, 2.0 MB. Copyright © 2021 Chrostek et al. 2021 Chrostek et al. https://creativecommons.org/licenses/by/4.0/ This content is distributed under the terms of the Creative Commons Attribution 4.0 International license.

In the above-described protocols, flies developed from egg to adult at a given temperature, and after collection they were aged for three more days at the same temperature before infection with viruses. To dissect at which of these stages, development and/or aging, temperature influences *Wolbachia* protection, we tested the four possible combinations of the two different temperatures for these two stages (Fig. S4). Developmental temperature strongly influenced *Wolbachia* protection, which was higher at 25°C (CHR = −1.16, *P < *0.001). Aging of the flies at 25°C also interacted with *Wolbachia* protection, but increased it only slightly compared to that with aging at 18°C (CHR = −0.431, *P = *0.011). Therefore, *Wolbachia* protection against viruses is mainly dependent on the temperature of development from egg to adult.

10.1128/mBio.02923-20.4 FIG S4 Developmental temperature determines *Wolbachia*-conferred antiviral protection. *Wolb^+^* and *Wolb^−^* flies, 50 per *Wolbachia* status per condition per replicate, were raised from egg to adult at 18°C or 25°C (Dev), collected at an age of 0 to 1 days, kept for 3 days at 18°C or 25°C (aging), pricked with DCV (10^8^ TCID_50_/ml), and placed at 18°C. Mortality was recorded daily. Three replicates of the same experiment are shown. Download FIG S4, TIF file, 1.1 MB. Copyright © 2021 Chrostek et al. 2021 Chrostek et al. https://creativecommons.org/licenses/by/4.0/ This content is distributed under the terms of the Creative Commons Attribution 4.0 International license.

**FIG 4:**
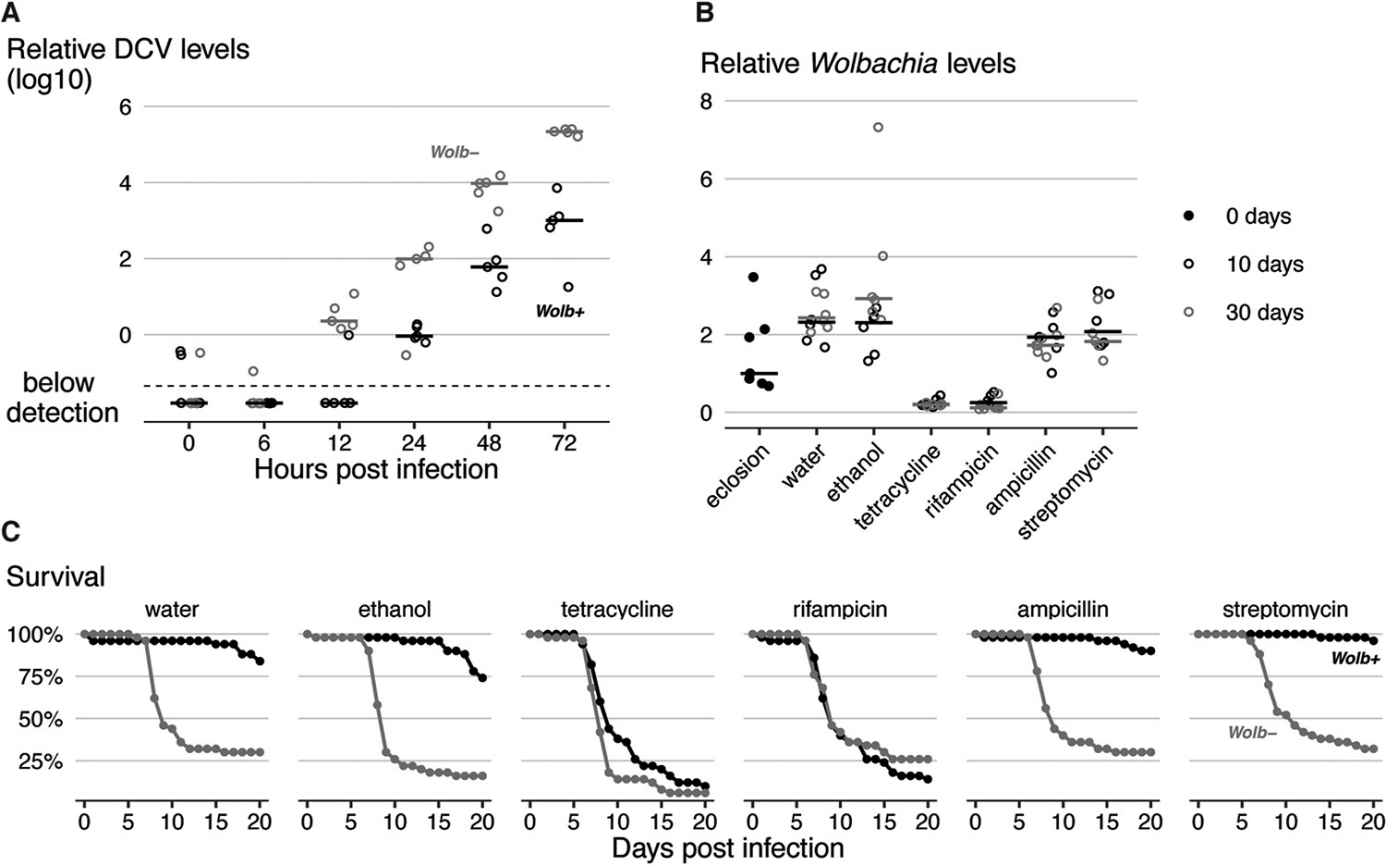
*Wolbachia* presence in adults is required for antiviral protection. (A) Time course analysis of DCV titers in *Wolb^+^* and *Wolb^−^* flies raised at 25°C and kept at 18°C after DCV infection. Relative DCV levels were determined by RT-qPCR. Time zero corresponds to the time of infection. Each dot represents a sample consisting of 10 flies; lines indicate medians. (B) *Wolbachia* levels in single flies raised at 25°C, at eclosion (day 0), after 10 days of different antibiotic and control treatments at 25°C (day 10), and after additional 20 days of treatment at 18°C (day 30), measured by qPCR. A replicate of this experiment is shown in Fig. S5C. (C) Survival of *Wolb^+^* and *Wolb^−^* flies, 50 per *Wolbachia* status per treatment, developed at 25°C, collected at eclosion, fed antibiotic-containing food for 10 days at 25°C, infected with DCV (10^8^ TCID_50_/ml), and placed at 18°C on antibiotic-containing food. Mortality was recorded daily. A replicate of this experiment is shown in Fig. S5E.

Since preinfection events are crucial for *Wolbachia*-conferred resistance, we asked how quickly the difference in virus titers arises between flies with and without *Wolbachia* (Fig. 4A). We observed a *Wolbachia*-induced resistance of approximately 120-fold as soon as we could detect viral RNA, at 12 h after infection, (LM, *P < *0.001 at 12 h; *P < *0.002 for all posterior time points in Fig. 4A and Fig. S5A). Thus, *Wolbachia* reduces viral titers very early in the course of DCV infection. We observed a similar pattern of early FHV blocking by *Wolbachia* from 1 day postinfection onwards, but with smaller viral titer differences (*Wolbachia*-infected flies had 2.3 to 5.5 times less FVH from the first day onwards; LM, *P < *0.028 for all these comparisons; Fig. S5B).

10.1128/mBio.02923-20.5 FIG S5 *Wolbachia* presence in adults is required for antiviral protection. (A) DCV titers in *Wolb^+^* and *Wolb^−^* flies raised at 25°C and kept at 18°C after DCV infection with two doses, 10^8^ and 10^9^ TCID_50_/ml, and at two time points post infection, 12 and 24 h. Relative DCV levels were determined by RT-qPCR. Each dot represents a sample consisting of 10 flies; lines show medians. (B) FHV titers in *Wolb^+^* and *Wolb^−^* flies raised at 25°C and kept at 25°C after infection. Each dot represents a sample consisting of 10 flies, lines indicate medians, and time zero corresponds to the time of infection. (C) *Wolbachia* levels in single flies raised at 25°C, at eclosion (day 0), after 10 days of different antibiotic/control treatments at 25°C (day 10), and after an additional 20 days of treatment at 18°C (day 30), measured by qPCR. This is a replicate of the experiment shown in Fig. 4B. (D) CFU counts in dissected *Wolb^+^* fly guts at day 30 of each antibiotic or control treatment. Each dot represents a pool of three guts. (E) Survival of *Wolb^+^* and *Wolb^−^* flies, 50 per *Wolbachia* status per treatment, developed at 25°C, collected at eclosion, fed antibiotic-containing food for 10 days at 25°C, infected with DCV (10^8^ TCID_50_/ml), and placed at 18°C on antibiotic-containing food. Mortality was recorded daily. This is a replicate of the experiment shown in Fig. 4C. Download FIG S5, TIF file, 1.7 MB. Copyright © 2021 Chrostek et al. 2021 Chrostek et al. https://creativecommons.org/licenses/by/4.0/ This content is distributed under the terms of the Creative Commons Attribution 4.0 International license.

Developmental temperature determines protection, and this protection can be detected as early as we detect viral replication in flies. Thus, we asked if changes in the fly caused by *Wolbachia* during development are sufficient to block viral infection in adults. To answer this, adult flies developed at 25°C were subsequently treated with antibiotics for 10 days to remove *Wolbachia* before viral infection (Fig. 4B and Fig. S5C). *Wolbachia* levels were reduced approximately 10-fold during 10 days of treatment with tetracycline and rifampicin (LMM, *P < *0.001 for both) and stayed low until the end of the experiment at 30 days (LMM, *P < *0.001 for both). These treatments eliminated antiviral protection from the flies (CHR, *P = *1.00 for both; Fig. 4C and Fig. S5E). The control treatments with the antibiotics ampicillin and streptomycin did not affect *Wolbachia* levels (LMM, *P > *0.254 for both; Fig. 4B and Fig. S5C) and did not affect endosymbiont-mediated protection (under both treatments, *Wolbachia* protection is significant [*P < *0.001] and not different from *Wolbachia* protection in controls [*P > *0.301]; CHR; Fig. 4C and Fig. S5E). As bacteria in the fly gut were efficiently cleared by all antibiotic treatments (Fig. S5D), we conclude that it is *Wolbachia* loss that leads to the loss of protection and not differential effect of the antibiotics on gut-associated bacteria. These data show that *Wolbachia* presence in adults is required for the *Wolbachia*-conferred antiviral protection, even though the presence or absence of protection is determined by the temperature during development, before the challenge occurs.

## DISCUSSION

Here, we show that temperature is a strong modulator of *Wolbachia*-conferred antiviral protection in the natural host D. melanogaster. The temperature at which the infection progresses influences this interaction, with *Wolbachia* giving more resistance and increasing survival at lower temperatures. However, the most striking phenotype we report is the effect of host developmental temperature on this interaction. While development at 25°C leads to a strong antiviral protection in terms of survival and resistance to DCV, development at 18°C abrogates or strongly reduces protection. This is observed with different genotypes of D. melanogaster, different variants of *Wolbachia* (*w*Mel and *w*MelCS), and different viruses, and is therefore likely to be a general phenomenon.

This complex interaction between temperature and *Wolbachia* protection against viruses may play a role in the natural environment. The outcome of *Wolbachia*-host interactions, in terms of antiviral protection, may differ in regions with different climates or across seasons in the same region. *Wolbachia* may provide more or less of a fitness benefit, depending on the conditions, and therefore the balance between the cost of harboring *Wolbachia* and the benefit may change with place and time. Our results lead to a prediction that *Wolbachia* would be more protective under warmer conditions, given the strong reduction in antiviral protection with a low developmental temperature. We also observed lesser protection at a higher temperature of infection, but this effect was weaker than the effect of temperature of development. We have suggested before that the antiviral protection is a fitness advantage that may explain the prevalence of *Wolbachia* in D. melanogaster populations (19). Selection for hosts carrying *Wolbachia* would, therefore, be stronger at higher temperatures. Consequently, geography and seasonality could impact the frequency of *Wolbachia* in a population. Interestingly, there is large variation in the frequency of *Wolbachia* in D. melanogaster natural populations and a clinal distribution of this frequency. At lower latitudes, and therefore in warmer climates, the frequency *of Wolbachia* is higher (45). However, the relationship between *Wolbachia* frequency, temperature and other environmental parameters in D. melanogaster is more complex at a smaller geographic scale, *Wolbachia* frequency is the highest in regions with a mean annual temperature of 22 to 26°C (53). In insects, in general, there is also a positive correlation between temperature and *Wolbachia* frequency, but only in temperate climates (50).

Despite ample laboratory data on *Wolbachia* protection against viruses in D. melanogaster, there is a lack of evidence for this in natural populations. A survey of presence of viruses in different D. melanogaster populations did not detect a correlation between frequencies of *Wolbachia* and different viruses (54). However, several reasons may have led to this lack of correlation, including lack of power to detect it. Moreover, the effect of *Wolbachia* on viruses may be more quantitative rather than be defined by presence/absence (see reference 54 for more). Moreover, it is difficult to predict what the correlation would be. Should *Wolbachia* presence be driven to high frequency in populations with a high frequency of viral infection, or should the frequency of viruses decrease in population with a high frequency of *Wolbachia*? The interaction between *Wolbachia* and viruses may lead to cyclic fluctuations and requires longitudinal sampling of natural populations to understand the relationships between *Wolbachia* and viruses in nature. A direct comparison of viral titers between *Wolbachia*-carrying and *Wolbachia*-free D. melanogaster flies from the same natural population could be an approach to test the impact of *Wolbachia* in nature. The comparison of these groups of flies from several locations in Australia did not detect an effect of *Wolbachia* on viral infection (55). However, viral presence and infection frequency assessments were performed in F1 flies raised in the lab at 19°C. This low temperature of development may have precluded the expression of the antiviral protection induced by *Wolbachia*. There is a need for further studies to understand the impact of *Wolbachia* on viruses in natural populations of D. melanogaster. Our results show that this impact may be very variable and strongly dependent on environmental conditions and may help to define how to test this effect in natural populations.

Other reasons may explain or contribute to clinal variation in *Wolbachia* frequency in natural populations. For instance, *w*Yak, which is closely related to *w*Mel, is imperfectly transmitted in Drosophila yakuba when flies are raised at a lower temperature (20°C versus 25°C), probably due to lower *Wolbachia* titers in these flies (44). This may explain why maternal transmission of *w*Yak in D. yakuba is lower in flies collected at higher altitudes than in those collected at lower altitudes. Also, *Wolbachia*-carrying D. melanogaster flies have lower fecundity and viability following dormancy induced by cold temperatures in laboratory experiments (45). On the other hand, cytoplasmic incompatibility decreases with higher temperature in Drosophila simulans males carrying *w*Ri (56), showing that temperature has different effects on *Wolbachia*-induced phenotypes in different host-*Wolbachia* strain combinations.

These results are also relevant for the deployment of *Wolbachia*-carrying mosquitoes to block dengue, Zika, chikungunya, and other arboviruses (21, 26, 57–59). It is already known that heat stress impacts *w*Mel in transinfected Aedes aegypti mosquitoes, reducing its titers (37, 39), that heatwaves can impact titers and frequency of *Wolbachia* in these mosquito populations (60), and that at low cycling temperatures *Wolbachia* titers decrease (61). On the other hand, temperatures varying between 25°C and 28°C do not seem to affect protection against viruses in this system (35). It would be important, however, to assess how lower temperatures influence *Wolbachia*-induced pathogen blocking in mosquitoes. Determining the temperature range of effectiveness of *Wolbachia*-deploying antiviral field interventions will be crucial to plan where to use them and when to combine them with other complementary approaches (e.g., insecticides).

Preinfection temperature has a drastic and enigmatic effect on *Wolbachia*-conferred antiviral protection. Lower temperatures during host development determine the level of protection in adult life. Understanding the molecular mechanism of this effect will elucidate how the environment interacts with host-microbe symbioses and may be key to understanding *Wolbachia*-conferred antiviral protection. The effect of developmental temperature acts solely on the interaction between virus and *Wolbachia* and does not affect viral infection by itself. The onset of the protection conferred by *Wolbachia* seems to be immediately or very soon after viral infection. We observed a difference in viral titers as soon as we detected viral replication in *Wolbachia*-free flies, 12 h after infection. Thus, *Wolbachia* likely inhibits the viral entry or first replication cycle *in vivo*. *Wolbachia* interference in early stages of viral infection is in agreement with cell culture data (62–64). However, this is not a simple preset antiviral state of the host, since *Wolbachia* is still required at the time of infection. The lower *Wolbachia* titers in flies that developed at 18°C, compared to those in flies that developed at 25°C, could partially explain a reduction in protection. However, this difference should not be enough to explain the complete lack of protection when flies develop at 18°C. We previously observed significant protection against DCV in flies carrying even lower titers of other *w*Mel variants (27). Nonetheless, tissue-specific differences in titers, set during development, may underlie the phenotypic differences. It would, therefore, be interesting to characterize in detail the spatial distribution of *Wolbachia* and virus in flies developed at the different temperatures. Comparative transcriptomic and metabolomic analysis of D. melanogaster and *Wolbachia* under these two conditions could elucidate not only how temperature affects protection, but also the mechanism of *Wolbachia* antiviral protection itself.

Temperature affects many insect-symbiont interactions and their phenotypes (1), including protective symbiosis (65–67). Therefore, this environmental factor may play a general critical role in determining the outcome of complex host-endosymbiont-pathogen interactions and shape the geographic distribution of insects and their symbionts. The phenotypic variation we report here, ranging from no protection to strong protection against viruses, indicates that temperature could be a crucial determinant of the cost-benefit ratio of carrying *Wolbachia*. Therefore, temperature could deeply impact the D. melanogaster-*Wolbachia* interaction in natural populations. Moreover, these results may have important implications for the deployment of *Wolbachia*-carrying mosquitoes to fight arbovirus transmission and may lead to new approaches to dissect the mechanism of *Wolbachia*-conferred antiviral protection.

## MATERIALS AND METHODS

### Fly strains and husbandry.

DrosDel *w*^1118^ *isogenic* D. melanogaster (*iso*) with *w*MelCS_b *Wolbachia* (*Wolb^+^*) and the matching control without *Wolbachia* (*Wolb^−^*) were described elsewhere (19, 27, 68). Aljezur 1 and W-20 D. melanogaster lines (*Wolb^+^* and *Wolb^−^*) were also described previously (19). We determined that the *Wolbachia* variants in both of these lines lack an IS*5* transposon insertion in gene WD1310, based on the primers described in Riegler et al. (69). This insertion is present in all *w*MelCS-like variants, but not in wMel variants (27, 69), and therefore Aljezur-1 and W-20 are both *w*Mel-like *Wolbachia* variants. Stocks were maintained at a constant temperature of 25°C on a diet consisting of: 45 g molasses, 75 g sugar, 70 g cornmeal, 20 g yeast extract, 10 g agar, 1,100 ml water, and 25 ml of 10% Nipagin, with the addition of live yeast (Sigma).

### Virus infection experiments.

DCV and FHV were produced and titrated in cell culture as described previously (19, 27). Flies for experiments were produced by placing 12 females and 6 males per bottle for 4 days to produce offspring at either 25°C, 18°C, or at fluctuating temperature (an 18°C to 25°C gradual increase over 12 h and a 25°C to 18°C decrease during the subsequent 12 h). After 10 days (25°C), 15 days (fluctuating temperature), or 20 days (18°C) the flies started to eclose. Unless otherwise specified, 0- to 3-day-old males were collected from the bottles and placed in the vials, 10 males per vial, on food without live yeast. Flies were aged for 3 more days at the developmental or otherwise indicated temperature. These 3- to 6-day-old flies were pricked intrathoracically with virus diluted in 50 mM Tris-HCl (pH 7.5). After infection, flies were placed at the indicated temperatures. Survival was monitored daily, and vials were changed every 5 days.

### Nucleic acids extractions and real-time qPCR.

DNA for the quantification of *Wolbachia* was extracted from pools of 10 flies using the DrosDel protocol (https://drosdel.org.uk/molecular_methods.php) (68) or from single flies with a protocol described previously (70). RNA for assessment of viral titers was extracted using TRIzol (Invitrogen), and cDNA was prepared using Moloney murine leukemia virus (MMLV) reverse transcriptase (Promega), as described previously (27). Real-time qPCRs were carried out in a 7900HT Fast real-time PCR system (Applied Biosystems) with the iQ SYBR green supermix (Bio Rad) or in a QuantStudio 7 Flex real-time PCR system (Applied Biosystems) with iTaq universal SYBR green supermix (Bio-Rad). *Wolbachia* was quantified using *wsp* as the target gene and *Drosophila Rpl32* as the reference gene. DCV was quantified with primers for DCV as the target gene and *Drosophila rpl32* as the reference gene. The primers used were as follows: *Wolbachia wsp*, 5′-CATTGGTGTTGGTGTTGGTG-3′ and 5′-ACCGAAATAACGAGCTCCAG-3′; DCV, 5′-TCATCGGTATGCACATTGCT-3′ and 5′-CGCATAACCATGCTCTTCTG-3′; FHV, 5′-ACCTCGATGGCAGGGTTT-3′ and 5′-CTTGAACCATGGCCTTTTG-3′; and *Drosophila rpl32*, 5′-CCGCTTCAAGGGACAGTATC-3′ and 5′-CAATCTCCTTGCGCTTCTTG-3′. The thermal cycling protocol for *Wolbachia* amplification was as follows: initial 50°C for 2 min, denaturation for 10 min at 95°C followed by 40 cycles of 30 s at 95°C, 1 min at 59°C, and 30 s at 72°C. For DCV and FHV, the same conditions were used, except for an annealing temperature of 56°C. Relative levels of *Wolbachia* or DCV were calculated by the Pfaffl method (71).

### Antibiotic treatment of flies.

*Wolb^+^* and *Wolb*^−^ flies were raised at 25°C, collected as 0- to 1-day-old adults, and placed on fly food with 100 mg/ml of tetracycline hydrochloride, rifampicin, ampicillin sodium, or streptomycin sulfate (all from Sigma) or control food with antibiotic solvent (water or ethanol). At day 10 of treatment, flies were challenged with DCV and placed at 18°C, and survival was followed for an additional 20 days. Food vials were changed every 3 days. At day 0, day 10, and day 30, flies were collected to assay *Wolbachia* levels by qPCR. At day 31, guts from three *Wolb^+^* flies per condition were dissected to assess the effect of antibiotics on the gut-associated bacteria. Guts were homogenized in 250 μl of sterile LB, and 30 μl was plated on mannitol agar plates. This medium sustains growth of the main gut-associated bacteria in lab D. melanogaster, Acetobacter, and Lactobacillus species (72). CFU were counted after 5 days of incubation at 26°C.

### Statistical analysis.

All of the statistical analysis was performed in R (73). The data sets and script of the statistical analysis and the output of this analysis are available from figshare (https://doi.org/10.6084/m9.figshare.13123271.v1) (64).

Analysis of survival data was performed with Cox proportional hazard mixed-effects models. Fixed effects, depending on the experiment, included temperature, dose of DCV, presence/absence of *Wolbachia*, and antibiotic treatment, while replicate vials within the same experiment or full experimental replicates were considered random effects. Model fitting was performed using the *coxme* package in R (74).

*Wolbachia* and DCV titers were analyzed with log-transformed qPCR data and linear models or general linear models. Model fitting was performed using *lm* or the *lme4* package in R (75).

The effect of interaction between factors in the models was determined by analysis of variance (ANOVA). *Post hoc* analysis of marginal (least-squares) means was used to compare between the conditions of interest using the *lsmeans* package in R (76).

Figures were produced using the *ggplot2* package (77).
