# Supplementary figures and images for: Wolbachia-Conferred Antiviral Protection Is Determined by Developmental Temperature
Source: mBio. 2021 Sep 7;12(5):e02923-20. doi: 10.1128/mBio.02923-20 (PMC8546536; doi:10.1128/mBio.02923-20)

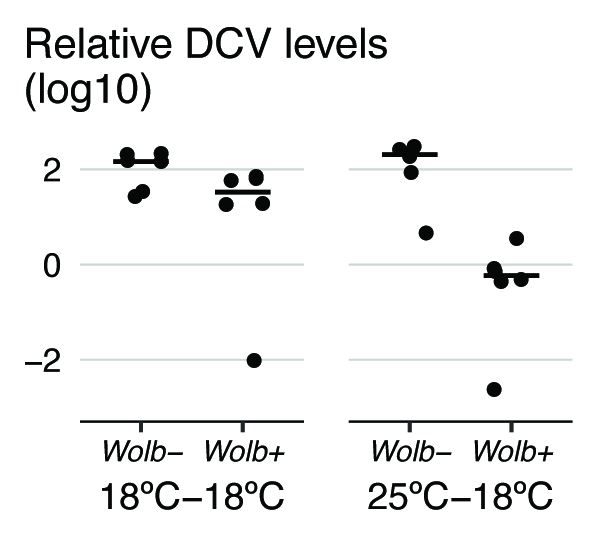

Supplement: FIG S1 [file mbio.02923-20-sf001.tif]

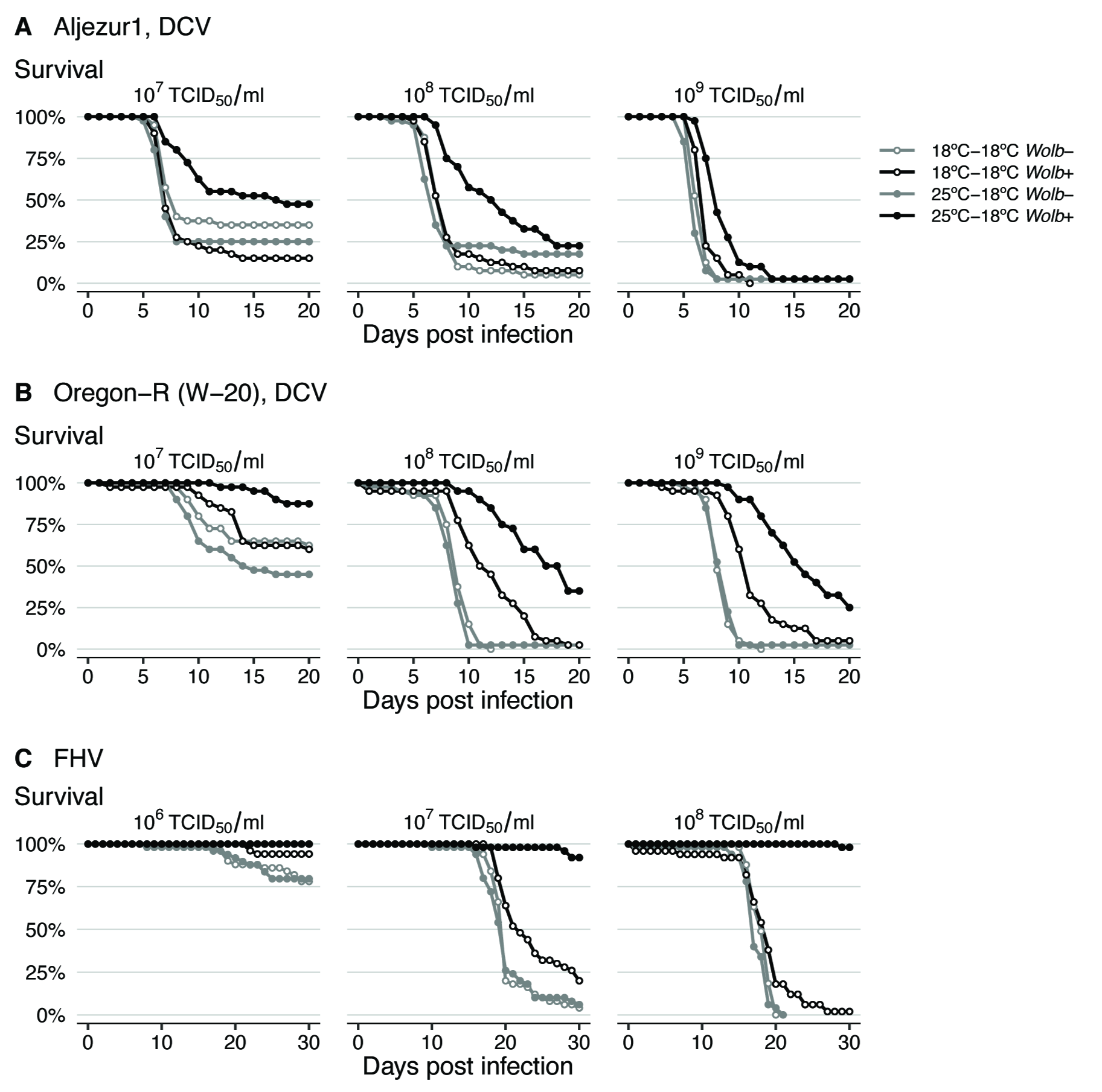

Supplement: FIG S2 [file mbio.02923-20-sf002.tif]

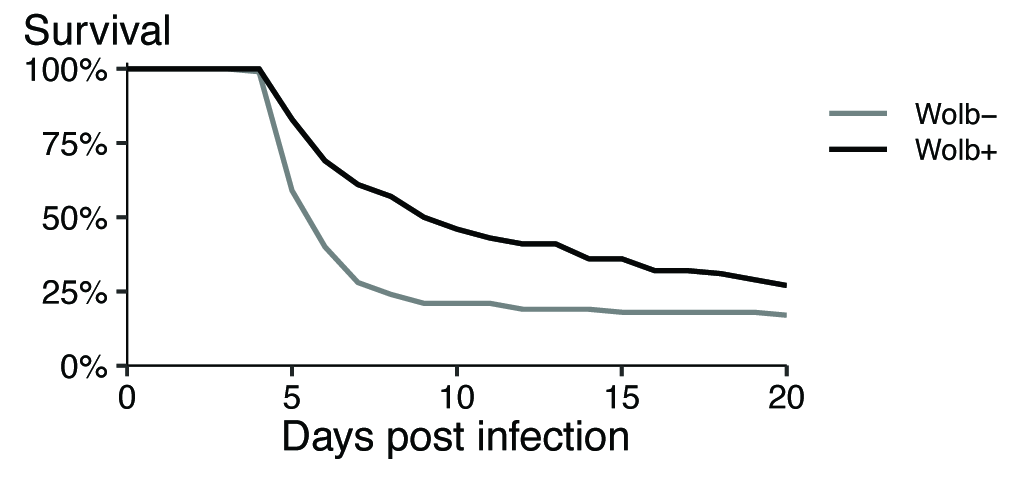

Supplement: FIG S3 [file mbio.02923-20-sf003.tif]

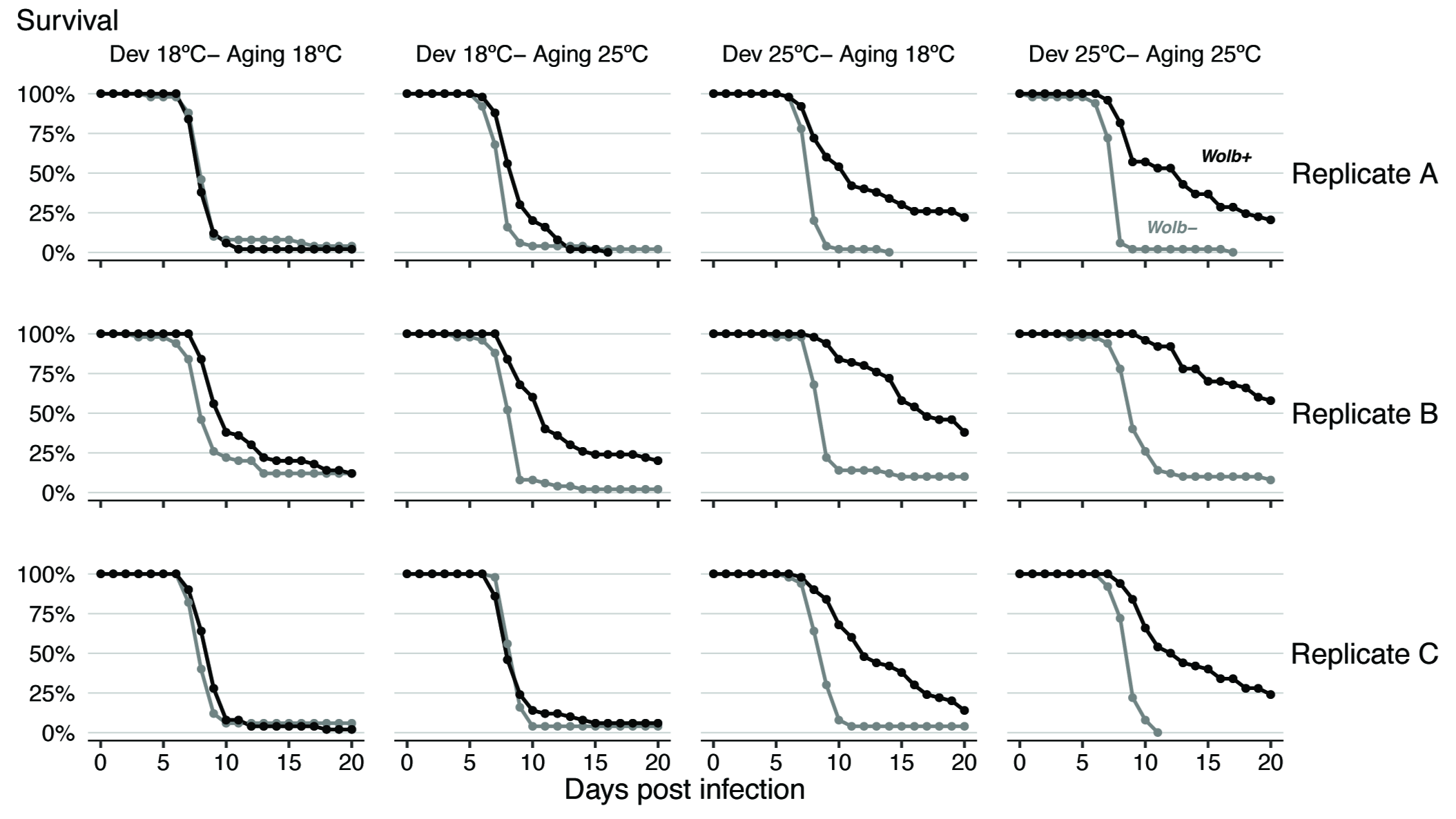

Supplement: FIG S4 [file mbio.02923-20-sf004.tif]

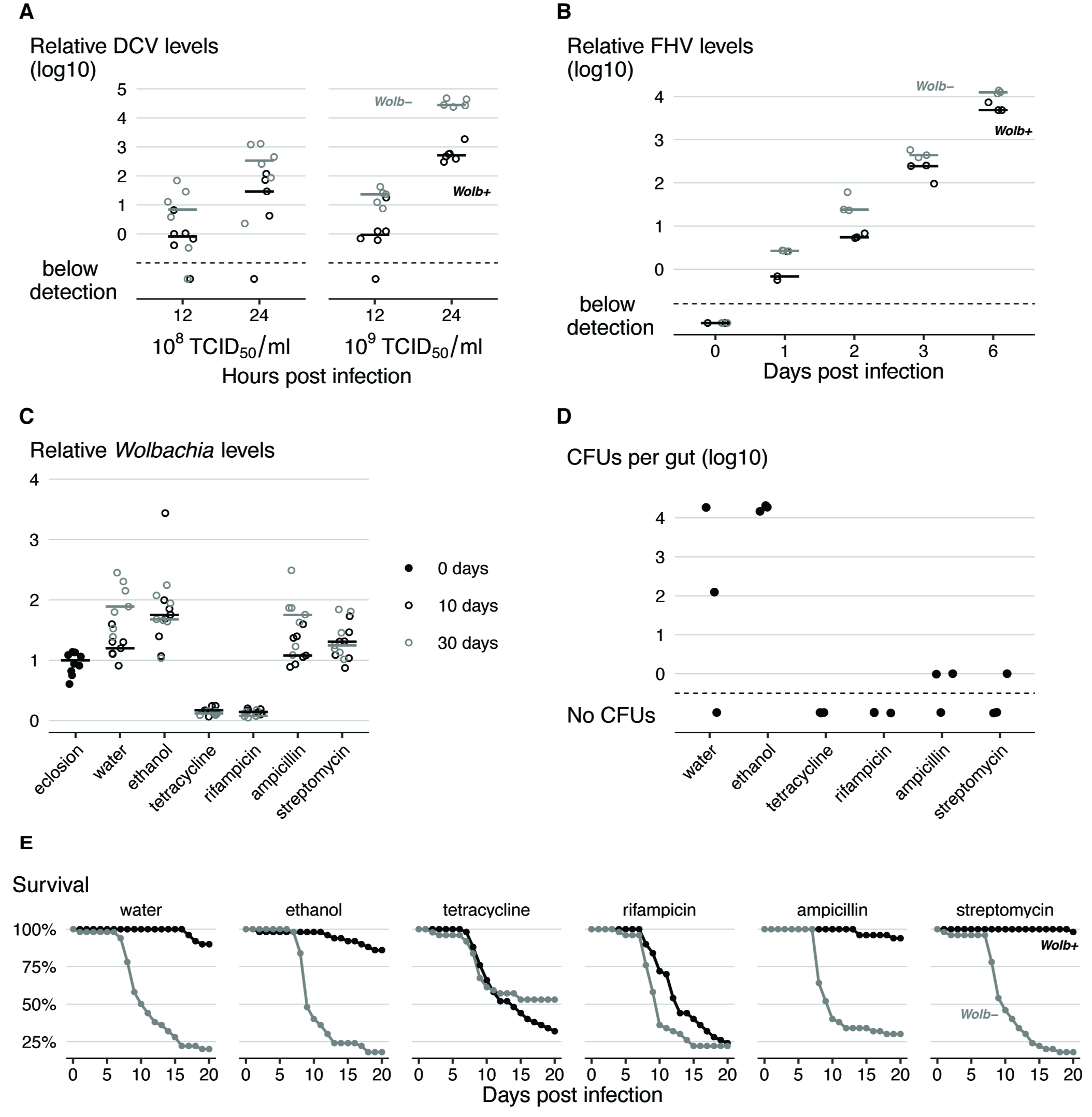

Supplement: FIG S5 [file mbio.02923-20-sf005.tif]
